# Carotid body tumor associated with complete heart block: A rare case report with long-term follow-up

**DOI:** 10.1016/j.ijscr.2025.111216

**Published:** 2025-03-27

**Authors:** Ahmad Hoseinzadeh, Hamed Nozari, Ebrahim Zeraatpisheh, Ali Tajaddini, Mohsen Mohebbiniya, Hamid Zaferani Arani

**Affiliations:** a Department of Surgery, Shiraz University of Medical Sciences, Shiraz, Iran; b Department of Pathology, Shiraz University of Medical Sciences, Shiraz, Iran

**Keywords:** Carotid body tumor, Complete heart block, Paraganglioma, Third-degree atrioventricular block

## Abstract

**Introduction:**

Carotid body tumor (CBT) is one of the most common paragangliomas in the head and neck region, accounting for only 0.6 % of tumors in this area. Here, we present a rare case of CBT associated with congenital complete heart block (CHB).

**Case presentation:**

We report the case of a 47-year-old female with a history of left neck and ear pain, accompanied by a gradually enlarging neck mass over 2.5 years. Following initial evaluations and multiple differential diagnoses, CT angiography revealed an oval-shaped mass measuring 30 × 50 mm at the left carotid bifurcation, confirming a Shamblin type II CBT. Preoperative assessments demonstrated congenital CHB on electrocardiogram and echocardiography. The patient underwent complete tumor resection with all necessary precautions, including temporary pacemaker placement and careful monitoring.

**Clinical discussion:**

CBTs are considered rare neoplasms, comprising only 0.6 % of head and neck tumors and approximately 0.03 % of all neoplasms. The surgical outcomes demonstrated that despite the coexistence of a CBT with CHB, safe and successful surgery is possible with careful planning and comprehensive management.

**Conclusion:**

This case highlights the importance of precise diagnosis and management in rare combination cases and emphasizes the crucial role of collaboration between vascular surgery and anesthesiology specialists in minimizing surgical risks and improving patient quality of life.

## Highlights

• Carotid body tumor is one of the most common paragangliomas in the head and neck region.
• We present a rare case of carotid body tumor associated with congenital complete heart block in a 47-year-old female.
• CT angiography revealed an oval-shaped mass measuring 30×50 mm at the left carotid bifurcation.
• The crucial role of collaboration between vascular surgery and anesthesiology specialists was presented in this case.

## 1 Introduction

Carotid body tumor (CBT), or glomus tumor, is the most common paraganglioma of the head and neck, originating from neural crest-derived chemoreceptor cells. These rare neoplasms, accounting for only 0.6 % of head and neck tumors, are often associated with chronic hypoxemia, such as in chronic obstructive pulmonary disease or high-altitude living, and exhibit a female predominance, typically presenting between ages 30 and 40 [1,2]. While most CBTs are benign, 2–8 % demonstrate malignant potential, and a subset may secrete catecholamines, complicating their clinical management [3,4]. The development of CBTs is influenced by genetic predisposition, environmental hypoxia, and hormonal factors, particularly estrogen, which has been linked to tumor growth acceleration [5].

Clinically, CBTs present as painless neck masses that, with progressive growth, compress surrounding structures, potentially invading the skull base or cranial nerves. Symptoms may include dysphagia, voice changes, Horner's syndrome, or vagal nerve compression, with diagnosis relying on imaging due to the high bleeding risk associated with biopsy [2,6]. The carotid sinus, a critical baroreceptor, regulates blood pressure and heart rate through parasympathetic pathways. In pathological conditions, CBTs can induce abnormal vagal stimulation, either through direct compression or neural invasion, leading to severe cardiovascular complications such as bradycardia, hypotension, sinus node arrest, or atrioventricular (AV) conduction disturbances [7]. Hence, in line with the SCARE criteria [8], this case highlights the rare coexistence of CBT with congenital CHB, emphasizing the potential for CBTs to exacerbate pre-existing cardiac conduction abnormalities through vagal interference.

## 2 Case presentation

A 47-year-old female with a history of congenital CHB presented with complaints of left-sided neck pain and left ear pain. Approximately 30 months prior to presentation, the patient had noticed a small mass on the left side of her neck, which had gradually increased in size. The pain was initially localized to the neck region but had intensified over time and extended to the left ear area.

**Fig. 1:**
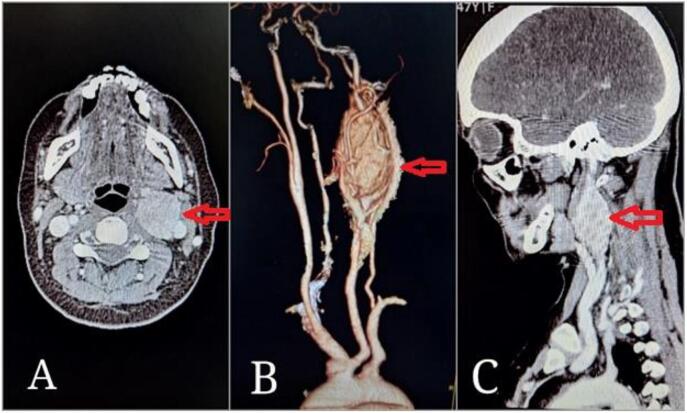
Pre-operative CT Angiography Images: Axial view (A), 3D reconstruction (B), and Sagittal view (C) (Red arrows).

Initially, due to left ear pain, the patient consulted several otolaryngologists and was treated for a preliminary diagnosis of otitis media. However, despite treatments, the pain persisted. Eventually, an otolaryngologist referred the patient for an ultrasound, which revealed a mass in the left side of the neck, leading to a surgical referral. After initial evaluations, a CT angiography was requested, which revealed an oval-shaped mass measuring 30 by 50 mm at the left carotid artery bifurcation, suggestive of a Shamblin type II CBT (Fig. 1).

### 2.1 Preoperative cardiovascular evaluations

**Fig. 2:**
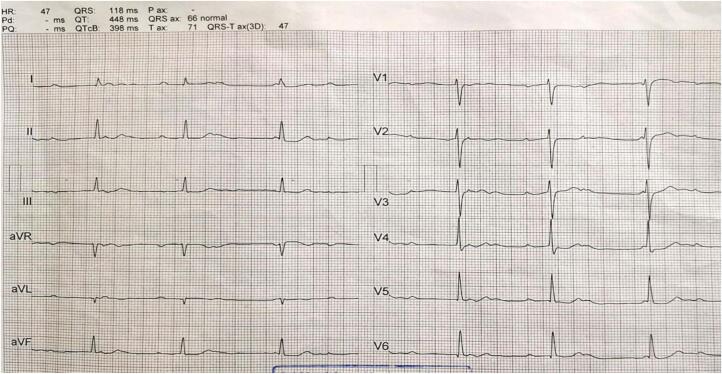
Patient's pre-operative ECG showing complete heart block.

Given the patient's history of congenital CHB, a comprehensive preoperative cardiovascular assessment was conducted. This included a 12‑lead electrocardiogram (ECG), which confirmed the presence of CHB with a stable ventricular escape rhythm (Fig. 2). Transthoracic echocardiography was performed to evaluate cardiac structure and function, revealing normal ventricular systolic function with no evidence of structural abnormalities. Additionally, a cardiology consultation was obtained to assess the patient's fitness for surgery and to optimize perioperative management.

### 2.2 Anesthetic considerations

**Fig. 3:**
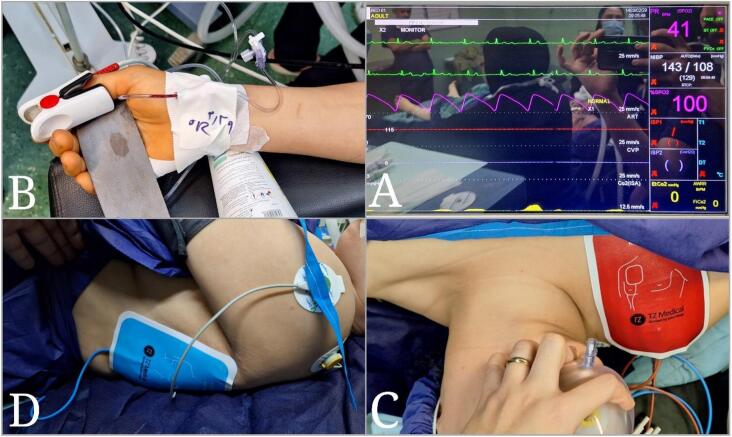
Images of patient preparation before surgery: Vital signs monitor (A), Arterial line (B), External pacemaker (C), External pacemaker (D).

Due to the patient's congenital CHB and the potential for hemodynamic instability during surgery, a detailed anesthetic plan was formulated. The patient was classified as American Society of Anesthesiologists (ASA) physical status III. Preoperatively, an external temporary pacemaker was placed to ensure adequate heart rate control, and an arterial catheter was inserted for continuous blood pressure monitoring (Fig. 3). Induction of anesthesia was performed using a combination of etomidate (0.3 mg/kg) and fentanyl (2 μg/kg) to minimize hemodynamic fluctuations. Maintenance was achieved with sevoflurane (1–2 %) and rocuronium (0.6 mg/kg) for muscle relaxation. Hemodynamic parameters, including heart rate, blood pressure, and oxygen saturation, were continuously monitored. Additionally, a central venous catheter was placed to monitor central venous pressure and administer vasoactive medications if needed. The anesthesia team maintained close communication with the surgical team to promptly address any intraoperative hemodynamic changes.

### 2.3 Surgical approach

The patient underwent surgery on May 19, 2024, after precise localization of the CBT via CT angiography. The surgical approach was chosen based on the Shamblin type II classification, indicating partial encasement of the carotid arteries. Given the tumor's proximity to critical neurovascular structures, including the vagus and hypoglossal nerves, an open surgical approach was deemed the most appropriate to ensure complete resection while minimizing complications.

**Fig. 4:**
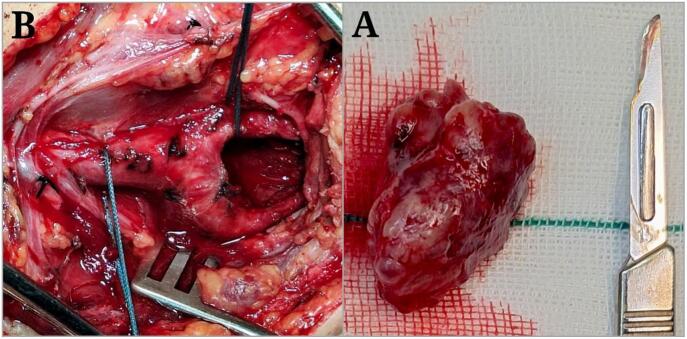
Images of the carotid body tumor: Resected CBT (A), Carotid artery bifurcation after CBT removal (B).

Following general anesthesia induction, the patient was positioned supine with the neck extended and rotated to the right. An oblique incision was made parallel to the left sternocleidomastoid muscle, extending from the mastoid process to the sternal notch. After skin incision and platysma dissection, the carotid sheath was carefully exposed. The common carotid artery and its bifurcation into the internal and external carotid arteries were identified. The tumor, measuring 30 × 50 mm, was observed at the carotid bifurcation and meticulously dissected from surrounding tissues (Fig. 4).

### 2.4 Specific maneuvers and precautions

1. Temporary vascular clips were applied to the internal and external carotid arteries to control blood flow during tumor dissection. This minimized bleeding and provided a clear surgical field.
2. Intraoperative neuromonitoring was used to assess the integrity of the vagus and hypoglossal nerves. Continuous feedback ensured that neural structures were preserved during dissection.
3. The tumor was carefully separated from the carotid arteries and surrounding neural structures using a combination of sharp and blunt dissection. Hemostasis was achieved using bipolar cautery and surgical clips to minimize bleeding.
4. The external pacemaker was actively monitored throughout the procedure to ensure stable heart rate and rhythm. The anesthesia team was prepared to administer atropine or epinephrine if significant bradycardia or hypotension occurred.

### 2.5 Postoperative management

After ensuring no active bleeding and reestablishing blood flow, tissues were returned to their original position, and the wound was closed in multiple layers. Appropriate dressing was applied. The surgery was completed without complications and with minimal bleeding in 2 h and 10 min. The patient was then transferred to the vascular ICU for close monitoring.

Postoperatively, the patient was monitored in the ICU for 24 h and later transferred to the general ward after clinical improvement. The external pacemaker was removed on postoperative day 1, as the patient maintained a stable ventricular escape rhythm. The patient was discharged in good general condition on postoperative day 3 without specific complaints.

### 2.6 Follow-up and long-term outcomes

**Fig. 5:**
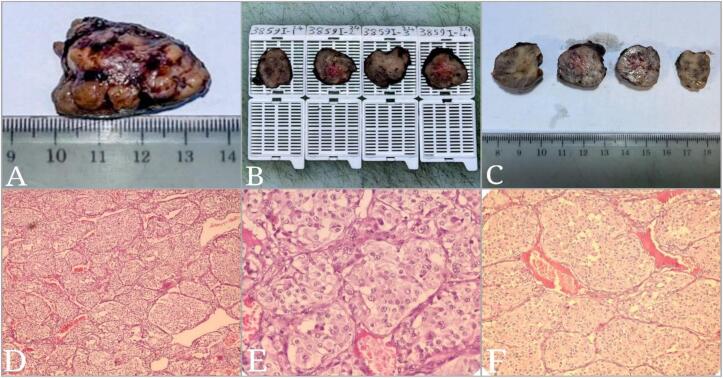
Pathology report images: Macroscopic views (A, B, C), Microscopic views (D, E, F).

**Fig. 6:**
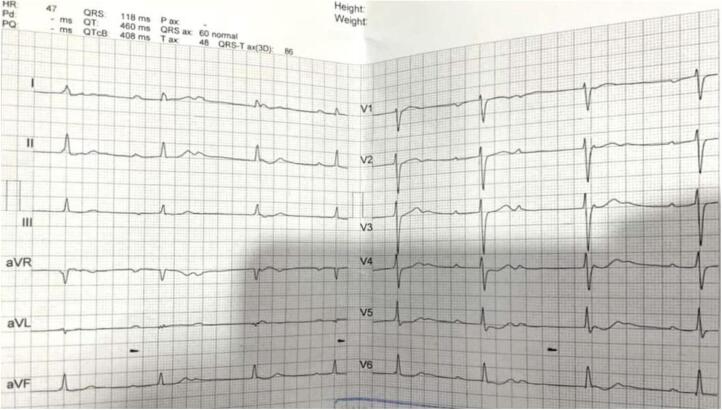
Patient's ECG 6 months after surgery: showing complete heart block.

Histopathological examination confirmed the definitive diagnosis of CBT in the patient (Fig. 5). Follow-up evaluations were conducted at 2 weeks, 1 month, 6 months, and 1 year post-surgery to assess both surgical and cardiac outcomes. At each visit, the patient underwent a 12‑lead ECG to monitor the congenital CHB, which remained unchanged throughout the follow-up period (Fig. 6). Transthoracic echocardiography was repeated at 6 months and one year, showing no deterioration in cardiac function and stable ventricular systolic function.

To evaluate the impact of tumor removal on cardiac function, heart rate variability (HRV) analysis was performed preoperatively and at 6 months post-surgery. Preoperative HRV analysis demonstrated reduced parasympathetic activity, likely due to vagal nerve compression by the tumor. Postoperative HRV analysis showed a significant improvement in parasympathetic tone, suggesting that tumor removal alleviated vagal nerve compression and restored normal autonomic function.

Additionally, the patient reported complete resolution of neck and ear pain following surgery, with no recurrence of symptoms during the follow-up period. Imaging studies, including Doppler ultrasound and CT angiography, were performed at 6 months and 1 year, confirming no evidence of tumor recurrence or vascular complications.

## 3 Discussion

This case highlights the rare coexistence of a CBT and congenital CHB, which holds significant clinical implications. CBTs, due to their anatomical proximity to the carotid sinus and vagus nerve, can exert mechanical pressure or cause neural invasion, leading to abnormal vagal stimulation. This can result in cardiovascular complications such as bradycardia, hypotension, or disturbances in cardiac conduction, particularly in patients with pre-existing cardiac conditions like CHB [4,6,7]. This case underscores the importance of multidisciplinary collaboration between vascular surgeons, cardiologists, and anesthesiologists in managing such complex cases. The preoperative placement of an external pacemaker and continuous hemodynamic monitoring were critical in mitigating risks during surgery. The successful outcome demonstrates that with meticulous planning, surgical intervention can be safely performed even in patients with significant cardiac comorbidities.

### 3.1 Congenital vs. acquired heart block

The pathophysiological connection between CBT and congenital CHB lies in the potential for vagal overstimulation [7]. The carotid sinus, a key baroreceptor, regulates heart rate and blood pressure through parasympathetic pathways. The congenital nature of the heart block in this patient was substantiated by the absence of acquired causes, such as infectious-inflammatory conditions or degenerative disorders, as confirmed by serological tests and echocardiography [9]. Also, the patient's stable ventricular escape rhythm and lack of structural abnormalities further support the congenital etiology. Indeed, in this patient, the tumor's location at the carotid bifurcation likely caused intermittent pressure on the vagus nerve, exacerbating the risk of hemodynamic instability. While the tumor did not directly cause the heart block, its presence likely exacerbated symptoms due to vagal nerve compression.

### 3.2 Comparison with previous literature

This case adds to the limited literature on CBTs associated with cardiac conduction disturbances. For instance, Gad et al. (2014) provides a comprehensive analysis of 56 CBTs managed over 25 years, emphasizing the importance of proper preoperative evaluation and surgical expertise in vascular reconstruction [10]. Similar to the current case, the study highlights the use of the Shamblin classification to guide surgical planning and reports a lower incidence of complications in *de novo* cases compared to those with prior surgical attempts. However, unlike the current case, which involved a rare coexistence of CBT with congenital CHB, they did not specifically address cardiovascular complications or the management of CBTs in patients with pre-existing cardiac conditions. Both studies underscore the critical role of multidisciplinary collaboration and advanced diagnostic tools in achieving favorable outcomes, with the current case further contributing to the literature by demonstrating the safe management of CBT in a high-risk cardiac patient.

In the another study, Darouassi et al., [11] analyzed 10 cases of CBTs over a 5-year period, emphasizing the importance of early diagnosis and multidisciplinary management. Similar to the current case, the study identified a slow-growing neck mass as the primary clinical presentation and relied on advanced imaging techniques, such as CT angiography, for diagnosis. Also, both studies underscore the critical role of surgical excision as the treatment of choice, with the referenced study also highlighting the use of preoperative embolization and postoperative radiotherapy in select cases. However, management(s) of cardiovascular complications in the patients with CBTs was not addressed [11].

### 3.3 Diagnostic and follow-up considerations

To exclude multiple lesions and confirm the non-secretory nature of the tumor, additional diagnostic studies were performed. An octreoscan and MRI were conducted, which confirmed the absence of multiple lesions. Plasma and urinary catecholamine levels were within normal limits, ruling out a secretory tumor.

Follow-up recommendations included annual imaging (CT angiography or MRI) for the first 5 years to monitor for tumor recurrence, along with regular cardiology evaluations to assess cardiac function. The patient was also advised to report any new symptoms promptly.

### 3.4 Limitations

This study has several limitations. The follow-up period of 1 year, while longer than initial reports, remains insufficient to assess long-term cardiovascular outcomes and tumor recurrence. Additionally, the lack of genetic testing to rule out familial paraganglioma syndromes is a notable limitation, as genetic predisposition could influence tumor behavior and recurrence risk. Future studies should include longer follow-up periods and genetic evaluations to provide more comprehensive insights.

## 4 Conclusion

This case report demonstrates that surgical resection of a CBT can be safely performed in patients with congenital CHB, provided that appropriate precautions are taken. The improvement in parasympathetic tone post-surgery highlights the potential cardiovascular benefits of tumor removal, while the 1-year follow-up data confirm the stability of cardiac function and the absence of tumor recurrence. This case underscores the importance of meticulous surgical planning as well as multidisciplinary collaboration in achieving optimal outcomes for complex cases.

## Patient consent

Written informed consent was obtained from the patient for publication and any accompanying images. A copy of the written consent is available for review by the Editor-in-Chief of this journal on request.

## Ethical approval

The patient provided written consent for this case report, which is presented in an anonymized format. According to the guidelines at our school (School of Medicine, Shiraz University of Medical Sciences, Shiraz, Iran), single case reports do not require separate ethical approval, as they do not contain identifying information and do not involve experimental treatment.

## Guarantor

Dr. Hamid Zaferani Arani

## Funding

Not applicable.

## Author contribution

Ahmad Hoseinzadeh: Conceptualization, Methodology, Software, writing, editing.

Ali Tajaddini, Hamed Nozari, Mohsen Mohebbiniya: Data curation, Writing- Original draft preparation.

Ebrahim Zeraatpishe: Data curation, Writing- Original draft preparation.

Hamid Zaferani Arani: Patient management, Supervision, Writing, editing

## Conflict of interest statement

The authors declare that they have no competing interests.

## Data Availability

The clinical documentation of the presented case cannot be made public due to the detailed identifiable information of the patient.
